# Prevalence of anxiety and associated risk factors among maternal and child health hospital female attenders: a cross-sectional multicenter study

**DOI:** 10.3389/fpsyg.2026.1699059

**Published:** 2026-02-10

**Authors:** Cheng Song, Jia Tang, Zhixin Shi, Shiru Tang, Zirui Wang, Guanlin Zhang, Siyuan Liu, Xu Chen, Yueping Song

**Affiliations:** 1 School of Population and Health, Renmin University of China, Beijing, China; 2 Center for Population and Development Studies, Renmin University of China, Beijing, China; 3 Bigdata and Responsible Artificial Intelligence for National Governance, Renmin University of China, Beijing, China; 4 Institute of Clinical Pharmacology, Peking University First Hospital, Beijing, China

**Keywords:** anxiety, healthcare access, healthcare knowledge, maternal health, mental health

## Abstract

**Background/Objectives:**

Maternal anxiety represents a substantial public health concern, particularly among women who are attending Maternal and Child Health (MCH) hospitals. This study investigated anxiety prevalence and risk factors among MCH hospital female attenders in Henan Province, a region demographically representative of national trends, during the peak winter medical demand of December 2024.

**Methods:**

A cross-sectional study using convenience sampling was implemented to recruit 745 maternal participants from 5 MCH hospitals (comprising three Grade A tertiary hospitals and two other hospitals) in Henan Province of China during December 2024. Maternal anxiety was assessed using the Generalized Anxiety Disorder 7-item scale (GAD-7). Knowledge regarding maternal and child healthcare and self-efficacy in accessing healthcare services were evaluated using the self-developed Maternal and Child Health Knowledge Scale (Cronbach’s *α* = 0.932) and the Self-Efficacy in Healthcare Access Scale (SEHAS) (Cronbach’s *α* = 0.964). Statistical analyses were performed using univariate and multivariate logistic regression models to determine the association between sociodemographic/clinical factors and anxiety symptoms.

**Results:**

The overall prevalence of anxiety was 30.5% (95% CI: 26.8–34.1%). Anxiety was significantly associated with hospital level, departmental affiliation, and participants’ knowledge regarding healthcare. Specifically, higher anxiety levels were found among females attending Grade A tertiary MCH hospitals versus other hospitals (Reference: Other hospital; Grade A tertiary hospital, AOR = 2.318, 95% CI = 1.580–3.402), and being in the Pediatric Department was a stronger risk factor than being in the Maternity Department (AOR = 0.501) or reproductive services (AOR = 0.584). Additionally, lower scores in maternal healthcare knowledge (AOR = 0.967, 95% CI = 0.940–0.995) and self-efficacy in healthcare access (AOR = 0.954, 95% CI = 0.913–0.997) were significantly correlated with increased anxiety symptoms.

**Conclusion:**

Our findings highlight a critical need for the integration of mental health screening and tailored interventions within maternal healthcare frameworks. Enhancing maternal health literacy, improving accessibility to psychological support, and developing specific interventions based on healthcare settings and departments are crucial for addressing anxiety and improving maternal health outcomes.

## 1 Introduction

Maternal anxiety is a significant complication that extends well beyond the perinatal period, impacting women across multiple stages of reproductive and child-rearing life. Multiple independent studies indicated that anxiety during the perinatal period can adversely affect maternal wellbeing and is increasingly linked to suboptimal child health outcomes including an elevated risk of neurodevelopmental disorders (Zhang et al., 2021; Zhang et al., 2023). While research has extensively documented anxiety among perinatal women in obstetric settings (Zhong et al., 2025; Nisar et al., 2025; Yang et al., 2025), far less is known about anxiety among women who utilize Maternal and Child Health (MCH) services beyond perinatal care.

In China, the MCH system functions as a comprehensive healthcare gateway, providing not only perinatal services but also routine gynecological examinations, fertility treatments, and pediatric care for children. Each of these clinical contexts introduces distinct stressors, whether related to diagnostic uncertainty, fertility pressures, or the ongoing management of a child’s health, all of which may initiate or amplify anxiety (Ahorsu et al., 2022; Lu et al., 2021; Zhu et al., 2021; Li et al., 2022). Nevertheless, mental health screening and support within MCH settings remain narrowly focused on the perinatal period, leaving a critical gap in understanding and addressing anxiety among the broader population of MCH service users. This gap is particularly salient in China, where systemic surveys of anxiety in non-perinatal MCH attendees are scarce, and associated social determinants are poorly understood (Zhu et al., 2025; Yang and Yu, 2023). The unaddressed anxiety can undermine both maternal mental health and the effectiveness of other health interventions, thereby affecting overall maternal and child wellbeing. Furthermore, anxiety within these complex medical environments is often exacerbated by a sense of unfamiliarity and helplessness. Limited health literacy and low self-efficacy in navigating the healthcare system can impair a woman’s ability to access information and manage care effectively, thereby acting as significant psychological drivers of anxiety. To address this, our study aims to characterize the anxiety level within this critical population in Henan province, China.

## 2 Methods

### 2.1 Ethical consideration and funding

This study was approved by the Ethics Review Committee of the School of Population and Health, Renmin University of China (approval date: 2024-11-28). All participants provided written informed consent prior to their inclusion. This work was supported by the Big Data and Responsible Artificial Intelligence for National Governance and Center for Population and Development Studies, Renmin University of China. The funder had no role in study design, data collection and analysis, decision to publish, or preparation of the manuscript.

### 2.2 Study design and participants

We conducted a cross-sectional study in Henan province, one of China’s most populous provinces with a demographic profile that closely mirrors the national average in terms of age-sex distribution, urban–rural composition, and socioeconomic gradients. Its healthcare system encompasses well-developed tertiary hospitals in urban centers as well as secondary and primary institutions in rural areas, representing the tiered structure of China’s MCH service network. Therefore, findings from Henan can offer insights with strong external validity for central China and provide a valuable reference for similar settings nationwide. Participants were recruited using a convenience sampling strategy from five MCH hospitals, stratified to represent different tiers of the healthcare system: one provincial-level, two municipal-level, and two county-level hospitals. Given the high outpatient volume during the peak winter season and limited onsite resources, convenience sampling was selected as the most feasible approach to ensure efficient data collection without disrupting clinical operations.

**Figure 1:**
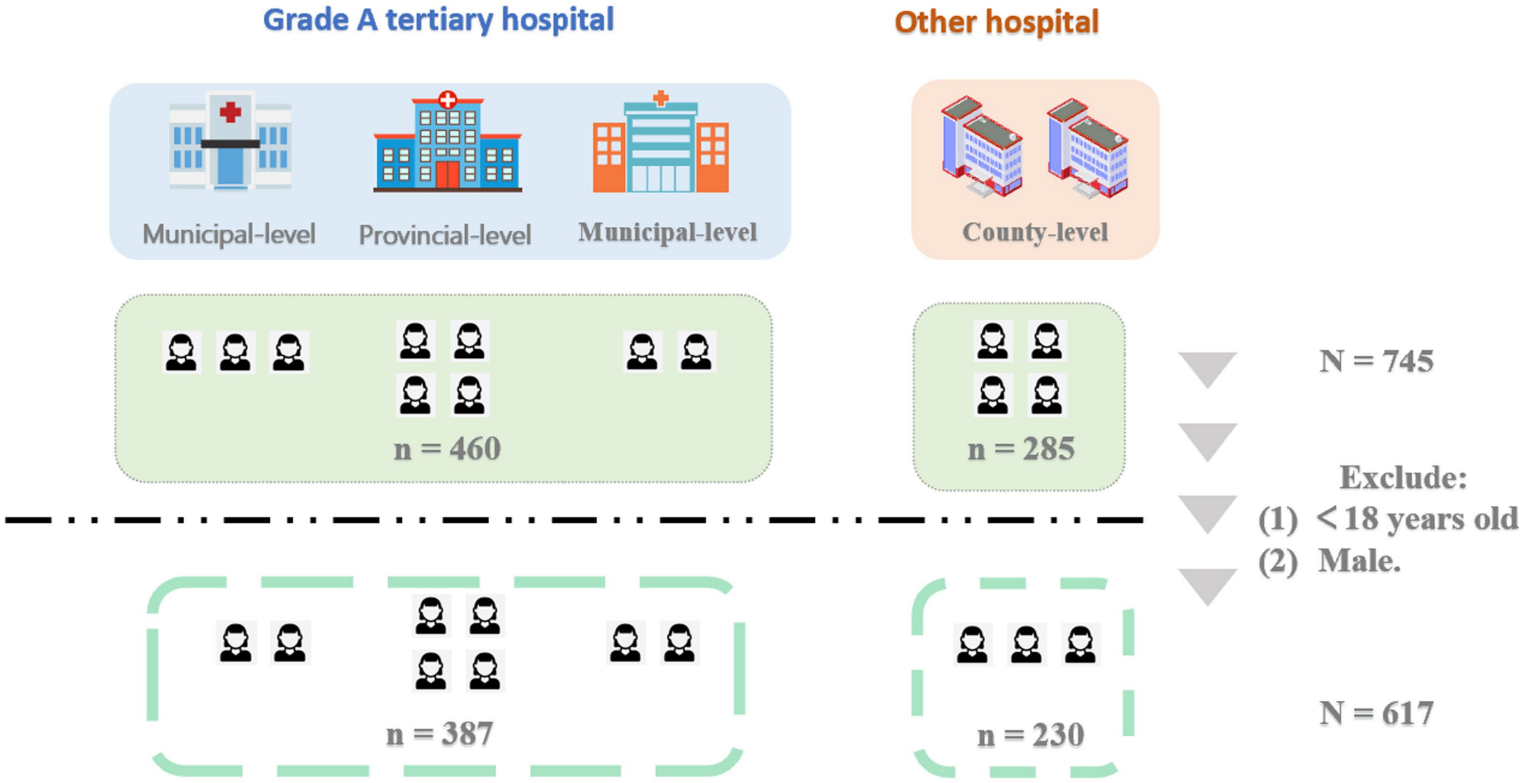
Flowchart of participant inclusion.

The inclusion criteria were defined as follows: (1) Biological females aged ≥18 years; (2) Current utilization of outpatient services in one of four designated departments: Maternity Healthcare (pregnant/postpartum women), Pediatric Healthcare (mothers or female guardians accompanying children), Gynecological Healthcare, or Reproductive Health Services; (3) Ability to communicate in Chinese and complete the questionnaire independently. A total of 745 participants completed the questionnaire. The following participants were excluded: (1) those who are under 18 years old, (2) male population. Ultimately, 617 participants were included in the final analysis of this study based on the above criteria (Figure 1).

### 2.3 Assessment tools

#### 2.3.1 Anxiety

Anxiety symptoms were assessed using the 7-item Generalized Anxiety Disorder scale (GAD-7) (Spitzer et al., 2006; Shih et al., 2022). Each item is rated on a 4-point Likert scale, from 0 (“not at all”) to 3 (“nearly every day”), with total scores ranging from 0 to 21. A score of ≥5 was used as the cutoff to indicate clinically significant anxiety symptoms, a threshold widely validated for screening purposes. In the present study, the GAD-7 demonstrated excellent internal consistency (Cronbach’s *α* = 0.945).

#### 2.3.2 Level of knowledge regarding maternal and child healthcare (knowledge)

The level of knowledge was measured by 6 items.

1. Attending community-based lectures on maternal and child healthcare within my residential neighborhood.
2. Participating in maternal and child healthcare seminars at hospitals or community health service centers.
3. Reviewing educational materials on maternal and child health distributed by the neighborhood committee.
4. Utilizing community- or street-level maternal and child health consultation services.
5. Examining maternal and child healthcare leaflets provided by hospitals or community health service centers.
6. Accessing and reading promotional materials on maternal and child health via online platforms (e.g., official websites, WeChat public accounts).

Each item was rated on a 5-point Likert scale, ranging from 1 (“never”) to 5 (“often”). A total score was calculated by summing the responses to all six items, with a possible range of 6 to 30. Higher scores indicate a greater frequency of proactive health information-seeking behavior. In the present study, this scale demonstrated excellent internal consistency (Cronbach’s *α* = 0.932).

#### 2.3.3 Self-efficacy in healthcare access (self-efficacy)

The level of Self-Efficacy in Healthcare Access (*SEHA*) was measured by 7 items.

1. I am knowledgeable about how to schedule prenatal examinations or book other MCH services.
2. I can comprehend the recommendations and guidance provided by healthcare professionals.
3. I am aware of which MCH services are reimbursable under my medical insurance.
4. Even when faced with certain challenges (e.g., time constraints, transportation barriers), I am capable of arranging appointments for MCH services.
5. With the support of physicians, I am confident in my ability to effectively address my health issues and attain the desired health outcomes.
6. I am able to adhere strictly to the follow-up schedules and health-management recommendations prescribed by my physician.
7. When selecting an MCH services provider, I proactively gather information and make the choice that best suits my needs.

Responses were captured on a 5-point Likert scale, from 1 (“Strongly disagree”) to 5 (“Strongly agree”). The total score was computed by summing the ratings for all seven items, yielding a potential range of 7 to 35. Higher scores reflect a stronger sense of self-efficacy in accessing and managing healthcare. This scale demonstrated excellent internal consistency in this sample (Cronbach’s *α* = 0.964).

#### 2.3.4 Covariates

We collected data on several demographic and clinical variables. Sociodemographic information include age (categorized as 18–44 vs. ≥45 years), marital status (married vs. unmarried/divorced/widowed), ethnicity (Han vs. other), education level (senior high school or below vs. above senior high school), and occupational status (employed vs. unemployed/retired).

Clinical and healthcare-related characteristics included self-reported health status (healthy vs. unhealthy) and hospitalization in the past year (yes vs. no). Information on the hospital level (tertiary vs. secondary) and the specific department visited (Maternity Healthcare, Pediatric Healthcare, Gynecological Healthcare, or Reproductive Health Services) was obtained from administrative records at the time of recruitment.

### 2.4 Statistical analysis

Descriptive statistics (frequencies, percentages, means, and standard deviations) were used to summarize participant characteristics. The primary outcome, anxiety, was treated as a binary variable (GAD-7 score ≥5 vs. <5).

We employed a two-step logistic regression modeling strategy. First, univariate logistic regression analyses were conducted to examine the unadjusted association between each potential predictor and anxiety. To avoid prematurely excluding potentially important variables, all factors with a *p*-value < 0.20 in the univariate analyses were selected as candidate variables for the multivariate model.

Second, a multivariate logistic regression model was built to identify independent factors associated with anxiety. The final model included the candidate variables identified in the first step. Results are presented as Odds Ratios (ORs) for univariate analyses and Adjusted Odds Ratios (AORs) for the multivariate analysis, both with 95% Confidence Intervals (CIs). A two-tailed p-value < 0.05 was considered statistically significant. All analyses were performed using R 4.2.1.

## 3 Results

### 3.1 Demographic characteristics of participants

Among 617 participants, 85.1% were married and 99.4% were identified as Han ethnicity. More than half of participants (54.8%) had received education beyond senior high school. Regarding employment status, 70.5% were currently employed, while 26.7% were unemployed and 2.8% were retired. In terms of health status, 83.8% of participants self-reported being in good health, and 17.1% had experienced hospitalization in the past year. Most participants (62.7%) received care at tertiary-level MCH hospitals, with the remaining 18.1% attending secondary-level hospitals. Participants were evenly distributed across four key departments: Maternity Healthcare (25.4%), Pediatric Healthcare (24.0%), Gynecological Healthcare (25.6%), and Reproductive Health Services (25.0%). This distribution reflects the diverse clinical services utilized by MCH services users within the MCH system. These demographic characteristics provide essential context for understanding the variability in anxiety levels and related risk factors observed in subsequent analyses (Tables 1, 2).

**Table 1:** Demographic characteristics of the enrolled women visiting MCH hospitals.

Characteristics	Number (%)
	*N* = 617
Age (years)	
18–44	529 (85.7)
≥45	88 (14.3)
Marital status	
Married	525 (85.10)
Unmarried/divorced/widowed	92 (14.90)
Ethnic groups	
Han	613 (99.4)
Other	4 (0.6)
Education level	
Senior high school or below	279 (45.2)
Above senior high school	338 (54.8)
Occupational status	
Unemployed	165 (26.7)
Retired	17 (2.8)
Employed	435 (70.5)
Self-reported health status	
Unhealth	100 (16.2)
Health	517 (83.8)
Hospitalization in the past year	
No hospitalization	511 (82.9)
Hospitalized	106 (17.1)
Hospital level	
Other hospital	230 (37.3)
Grade A tertiary hospital	387 (62.7)
Specific departments	
Maternity healthcare department	157 (25.4)
Pediatric healthcare department	148 (24.0)
Gynecological healthcare department	158 (25.6)
Reproductive health services department	154 (25.0)

**Table 2:** Mental health status of the enrolled women visiting MCH hospitals.

Variable	Number (%)	Mean (SD)	Range
	*N* = 617		
Anxiety			
Yes	188 (30.5)		
No	429 (69.5)		
Anxiety			
Total score		3.14 (3.71)	0–21
Knowledge			
Total score		20.13 (6.95)	6–30
SEHA			
Total score		31.46 (4.23)	7–35

### 3.2 Univariate analysis of demographic and anxiety in participants

The results of univariate analysis of anxiety were shown in Table 3. The factors associated with anxiety among participants were: hospital level (Grade A tertiary hospital, OR = 2.318, 95% CI = 0.294–0.633), specific departments (maternity healthcare department, OR = 0.531 95% CI = 0.326–0.864; reproductive health services department, OR = 0.601, 95% CI = 0.371–0.974), knowledge (OR = 0.956, 95% CI = 0.932–0.980) and self-efficacy (OR = 0.947, 95% CI = 0.910–0.985).

**Table 3:** Univariate statistical analysis of anxiety for the enrolled women visiting MCH hospitals.

Characteristics	Anxiety	
	OR (95%CI)	*p* value
Age (years)		
18–44	0.929 (0.572–1.510)	0.767
≥45	1	
Marital status		
Married	0.890 (0.554–1.429)	0.629
Unmarried/divorced/widowed	1	
Ethnic group**s**		
Han	1.317 (0.136–12.743)	0.812
Other	1	
Education level		
Senior high school or below	0.780 (0.551–1.103)	0.160
Above senior high school	1	
Occupational status		
Unemployed	0.758 (0.509–1.130)	0.174
Retired	0.447 (0.126–1.580)	0.211
Employed	1	
Self-reported health status		
Unhealth	0.843 (0.505–1.407)	0.514
Health	1	
Hospitalization in the past year		
No hospitalization	0.790 (0.491–1.272)	0.332
Hospitalized	1	
Hospital level		
Grade A tertiary hospital	2.318 (1.580–3.402)	0.001^*^
Other hospital	1	
Specific departments		
Maternity healthcare department	0.531 (0.326–0.864)	0.011^*^
Reproductive health services department	0.601 (0.371–0.974)	0.039^*^
Gynecological healthcare department	0.657 (0.409–1.056)	0.083
Pediatric healthcare department	1	
Knowledge	0.956 (0.932–0.980)	0.001^*^
SEHA	0.947 (0.910–0.985)	0.007^*^

^*^p < 0.05.

### 3.3 Multivariate analysis of demographic and anxiety in participants

The results of univariate analysis of anxiety were shown in Table 3. Service users in Tertiary Class A hospitals showed higher vulnerability to anxiety symptoms than their counterparts in other tertiary hospitals (AOR = 0.439 for other tertiary hospitals; 95% CI = 0.292–0.660). Additionally, being in the Paediatric Department was a stronger risk factor than being in the Maternity Department (AOR = 0.501) or reproductive services (AOR = 0.584). Higher knowledge (AOR = 0.967, 95% CI = 0.940–0.995) and self-efficacy (AOR = 0.954, 95% CI = 0.913–0.997) showed the protective factor to anxiety symptoms (Table 4).

**Table 4:** Multivariate statistical analysis of anxiety for the enrolled women visiting MCH hospitals.

Characteristics	Anxiety	
	AOR (95%CI)	*p* value
Education level		
Senior high school or below	0.968 (0.639–1.467)	0.878
Above senior high school	1	
Occupational status		
Unemployed	0.788 (0.494–1.257)	0.317
Retired	0.331 (0.088–1.249)	0.103
Employed	1	
Hospital level		
Grade A tertiary hospital	2.278 (1.515–3.425)	0.001^*^
Other hospital	1	
Specific departments		
Maternity healthcare department	0.501 (0.302–0.830)	0.007^*^
Reproductive health services department	0.584 (0.355–0.961)	0.034^*^
Gynecological healthcare department	0.683 (0.417–1.118)	0.129
Pediatric healthcare department	1	
Knowledge	0.967 (0.940–0.995)	0.021^*^
SEHA	0.954 (0.913–0.997)	0.037^*^

^*^p < 0.05, adjustment was made for covariates demonstrating *p* < 0.20 in univariate analyses.

## 4 Discussion

This study provides a unique insight into the prevalence and determinants of anxiety symptom among women utilizing MCH services in Henan, China. By moving beyond the traditional focus on the perinatal period, our findings provide a reference for the mental health landscape of women across various stages of reproductive and maternal life. We identified a substantial prevalence of anxiety symptom and, more importantly, uncovered key modifiable factors related to both healthcare system structure and individual patient behaviors that are associated with this burden. Existing research has predominantly focused on specific populations such as pregnant and postpartum women, with a notable dearth of studies investigating the clinically significant group of MCH services users in MCH hospital (Lähdepuro et al., 2024; Nolvi et al., 2019; Nath et al., 2019). Our research effectively bridges this gap, intending to offer valuable insight for clinical practice.

Our findings reveal a considerable anxiety symptom prevalence rate of 30.5% (95% CI: 26.8–34.1%) among MCH services users who attended medical consultations at MCH hospitals in Henan province, underscoring a substantial mental health concern within this population. Given that previous studies have primarily focused on perinatal anxiety rather than the broader MCH population, direct comparisons are challenging. However, this 30.5% prevalence rate suggests that the mental health burden in this mixed clinical setting—which encompasses not only pregnancy-related stress but also the anxiety associated with pediatric illness and gynecological conditions—is substantial and warrants urgent attention.

A key finding of our research is the significant variation in anxiety symptom levels across different level MCHs. Women attending grade A tertiary hospitals reported significantly higher anxiety symptom than those at other hospitals. This may reflect the differences in caseload complexity, patients’ expectation, or the institutional environment itself among grade A tertiary hospitals and other hospitals. Grade A tertiary hospitals often manage more severe or complex cases, which can create a more stressful atmosphere for patients. In China, these top-tier facilities are frequently characterized by overcrowding and intricate referral bureaucracies. Patients often endure long queues only to receive brief consultation sessions, leading to a rushed and impersonal care experience. This result suggests that strengthening mental health support at the tertiary level, where the clinical and psychological stakes are often highest, is a critical priority.

Furthermore, our data revealed that women visiting the pediatric department experienced the highest levels of anxiety symptom. This finding is consistent with literature underscoring the profound psychological toll that a child’s health issues can exert on a mother (Ueki et al., 2015). The acute, often uncontrollable, nature of pediatric health concerns appears to be a more potent driver of anxiety symptom than the challenges faced in maternity or reproductive health settings. This powerfully argues for embedding maternal mental health support directly within pediatric care models, recognizing that a mother’s wellbeing is inextricably linked to that of her child (Avan et al., 2023; Ueki et al., 2014).

Perhaps our most actionable finding relates to the individual-level protective factors we identified. We found that more frequent engagement in health information-seeking behaviors and higher self-efficacy in accessing healthcare were both independently associated with a lower likelihood of anxiety symptom. This moves beyond the simplistic notion that merely “knowing more” reduces anxiety symptom. Instead, it suggests that the proactive process of seeking information is a key component of an adaptive coping strategy. By actively engaging with health resources, women may enhance their sense of control, improve their ability to navigate complex healthcare systems, and build confidence in their decision-making—all of which are potent buffers against anxiety symptom (Zhu et al., 2025). Specifically, improving ‘navigational’ self-efficacy can reduce the ‘learned helplessness’ often felt in large hospitals. This finding has direct implications for intervention design: efforts should not only focus on disseminating information but also on empowering women by lowering barriers to access and cultivating the skills and confidence needed to become active participants in their own healthcare (Liu et al., 2023; Shephard et al., 2019).

The distinct patterns of anxiety symptom identified across different clinical settings and individual characteristics underscore the urgent need for a more integrated approach to mental health within routine MCH care. Our findings strongly suggest that, despite considerable progress in China’s healthcare coverage and accessibility, maternal mental health services remain insufficiently developed and underutilized. Previous studies conducted in high-income countries have consistently demonstrated the benefits of early detection and treatment of maternal anxiety, emphasizing that anxiety management during the antenatal and postnatal periods is crucial in improving both maternal and infant health outcomes (Laurenzi et al., 2020). Evidence from longitudinal studies highlights that untreated maternal anxiety can lead to complications such as preterm birth, low birth weight, postpartum depression, and developmental impairments in children (Galler et al., 2004; Padovani et al., 2011). Our findings reinforce these observations, emphasizing the need for systematic screening programs within antenatal care frameworks to proactively identify and manage anxiety.

Several limitations of this study should be acknowledged when interpreting the findings. First, the cross-sectional design inherently precludes the establishment of causal relationships; while we identified strong associations, we cannot determine whether, for instance, lower self-efficacy leads to anxiety symptom or if anxiety symptom erodes an individual’s self-efficacy over time. Future research should employ longitudinal or prospective cohort designs to delineate these temporal and potentially bidirectional pathways. Second, our reliance on a convenience sampling strategy, although geographically stratified across different hospital tiers, may limit the generalizability of our prevalence estimates to the entire population of Henan province. Future studies using probability-based sampling methods would provide more robust population-level estimates. Third, all key measures, including the primary outcome of anxiety symptom, were based on self-report questionnaires, which are susceptible to social desirability and recall biases. While the GAD-7 is a well-validated tool, it does not replace a formal clinical diagnosis. The inclusion of objective clinical assessments or a two-stage screening design in future research could yield more precise diagnostic information. Fourth, our self-developed scales for information-seeking behavior and self-efficacy, while demonstrating excellent internal consistency, have not yet undergone formal external validation or test–retest reliability analysis. Further psychometric evaluation of these instruments in diverse populations is warranted. Finally, while our multivariate model adjusted for several key demographic and clinical factors, the possibility of residual confounding from unmeasured variables, such as socioeconomic status, social support, or personal psychiatric history, cannot be entirely ruled out. Future investigations should aim to incorporate a more comprehensive set of potential confounders to provide a more nuanced understanding of the determinants of maternal anxiety symptom.

## 5 Conclusion

In conclusion, this study highlights that anxiety symptom is a significant public health concern among the diverse population of women served by MCH hospitals in China, particularly within Pediatric Departments and Grade A tertiary hospitals. The identification of key modifiable risk factors, including the hospital level, clinical department, and individual factors such as woman’s confidence and proactivity in engaging with the healthcare system. Our findings strongly advocate for the integration of accessible mental health services within existing frameworks, specifically recommending that maternal anxiety screening be conducted during pediatric consultations. Additionally, increasing support for hospital navigation is essential to boost women’s confidence. By addressing these identified risk factors through evidence-based policy interventions and refined clinical practices, guided by both our findings and global best practices, there is immense potential to improve maternal mental health outcomes and, consequently, enhance overall maternal wellbeing across the region and beyond.

## Data Availability

The raw data supporting the conclusions of this article will be made available by the authors, without undue reservation.
